# Supplementary figures and images for: Efficacy of novel SARS-CoV-2 rapid antigen tests in the era of omicron outbreak
Source: PLoS One. 2023 Aug 10;18(8):e0289990. doi: 10.1371/journal.pone.0289990 (PMC10414561; doi:10.1371/journal.pone.0289990)

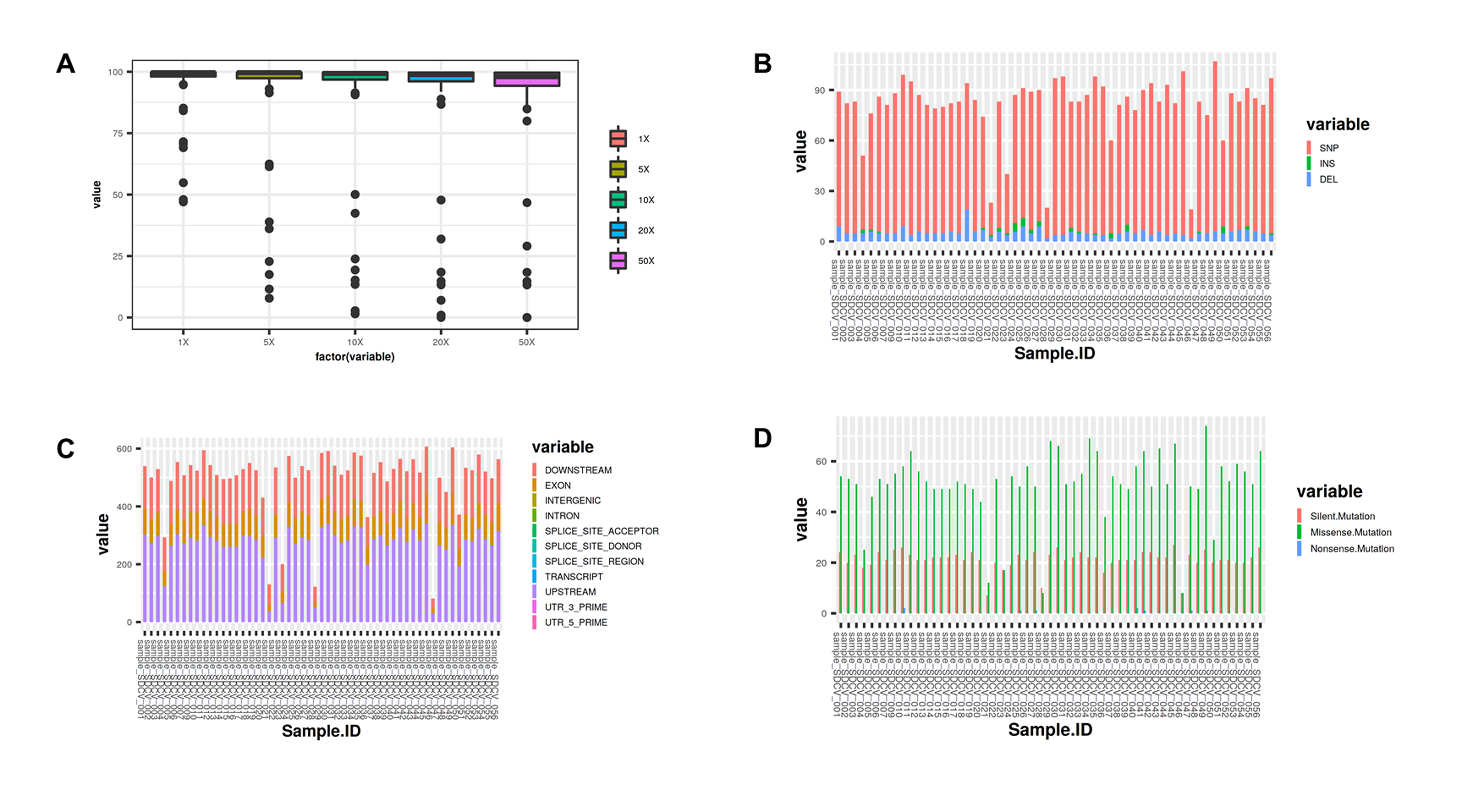

Supplement: S1 Fig — In, (A) the coverage depth of filtered raw data across reference sequences, (B) annotated variants that occur in the obtained sequences, (C) annotated categories of functionally important regions, and (D) mutation classes of sequence variants. (TIF) [file pone.0289990.s001.tif]
