# Supplementary material for: Efficacy of novel SARS-CoV-2 rapid antigen tests in the era of omicron outbreak
Source: PLoS One. 2023 Aug 10;18(8):e0289990. doi: 10.1371/journal.pone.0289990 (PMC10414561; doi:10.1371/journal.pone.0289990)
Supplement: Attachment — Submitted filename: Response to Reviewers.docx [file pone.0289990.s002.docx]

# The authors would like to thank the Reviewers for their specific and helpful comments on the manuscript. The authors have carefully taken the comments into consideration and have made revisions to the manuscript to address reviewers’ and editors’ concerns.

Responses to the Reviewer are reported as follows:

# Journal requirements:

1. Please ensure that your manuscript meets PLOS ONE's style requirements, including those for file naming. The PLOS ONE style templates can be found at

https://journals.plos.org/plosone/s/file?id=wjVg/PLOSOne_formatting_sample_main_body.pdf and 
https://journals.plos.org/plosone/s/file?id=ba62/PLOSOne_formatting_sample_title_authors_affiliations.pdf

**Response:**

We have ensured that the file naming has met PLOS ONE's style requirements

2. We note that you have included the phrase “data not shown” in your manuscript. Unfortunately, this does not meet our data-sharing requirements. PLOS does not permit references to inaccessible data. We require that authors provide all relevant data within the paper, Supporting Information files, or in an acceptable, public repository. Please add a citation to support this phrase or upload the data that corresponds with these findings to a stable repository (such as Figshare or Dryad) and provide URLs, DOIs, or accession numbers that may be used to access these data. Or, if the data are not a core part of the research being presented in your study, we ask that you remove the phrase that refers to these data.

**Response:**

We have added the mentioned data to Table 2.

**Pages 11-12, Lines: 219-225, Table 2 (*E* gene)**

**Reviewers Comments to the Author**

**Reviewer #1:**

The current manuscript is strict-forward in determining the sensitivity of SARS-CoV-2 Rapid Antigen Test 2.0 and SARS-CoV-2 Rapid Antigen Test 2.0 Nasal to Omicron variant. Not many major concerns exist. Some clarification and elaboration can improve the manuscript before publication. Thank you.

1. It will be better to mention earlier that SARS-CoV-2 Rapid Antigen Test 2.0 and SARS-CoV-2 Rapid Antigen Test 2.0 Nasal are designed and intended to detect the Omicron variant. This background information is unclear until line 294. What design was made for these two kits? E.g. specifying what specific detection antibodies are included in the kit will help improve the delivered knowledge of the paper.

**Response:**

We have confirmed to the manufacturer that SARS-CoV-2 Rapid Antigen Test 2.0 and SARS-CoV-2 Rapid Antigen Test 2.0 Nasal are not specifically designed for only detecting the Omicron variant but for all SARS-CoV-2 variants. However, given that there are some modifications, the manufacturer claimed that the antibodies have a better ability to capture the SARS-CoV-2 antigen regardless of the variant.

We have added this information on **pages 4-5 lines: 81-92.**

“In October 2022, Roche Diagnostics launched SARS-CoV-2 rapid antigen tests with an enhanced ability of antibodies in capturing the antigen, hence believed to be more effective in the detection of SARS-CoV-2 regardless of the variants.”

We also have modified the sentences in **line 294** to avoid any misunderstanding regarding the RATs.

**Page 16, lines 309-312.**

“In this study, we assessed the clinical sensitivity of novel SARS-CoV-2 RATs for the detection of SARS-CoV-2, particularly Omicron and its subvariants, from the nasopharyngeal (SARS-CoV-2 Rapid Antigen Test 2.0) and nasal swabs specimens (SARS-CoV-2 Rapid Antigen Test 2.0 Nasal).”

2. Please clarify the definition of “uncertain” in Table 4. Does it mean it is not Omicron, uncertain to be SARS-CoV-2 or negative?

**Response:**

We have added the definition of “**uncertain” in Table 4 legends.**

**“**Uncertain refers to the samples identified as SARS-CoV-2 Omicron variant but difficult to determine into accurate subvariant.”

3. Analysis method of WGS was not mentioned clearly (lines 147-161). What are the criteria for WGS to define Omicron subtypes and uncertain?

**Response:**

The Omicron subvariant was determined according to the mutations that occur in the sequence after comparing the sequence with the database in GISAID. In short, the obtained sequences were compared with the sequence data that were stored in the GISAID, and the mutation database was constructed for classifying the mutation that occurs. The Omicron subvariants were determined through variant calling in the WGS analysis. The result is referred to as “uncertain” when the lineage-specific variants are not detected, and only mutations shared by two or more lineages are called from the “variant calling”.

**Page 8, Lines: 169-174.**

4. What is the significance of having a RAT kit to detect specifically Omicron variant? Strengths and drawbacks can be discussed. How about a pan-SARS-CoV-2 strains RAT kit e.g. DIAGNOVIR (PMID: 36932107)? Possible and necessary to include a few more detection lines on a RAT kit to distinguish Omicron variants?

**Response:**

We have clarified that the SARS-CoV-2 Rapid Antigen Test 2.0 and SARS-CoV-2 Rapid Antigen Test 2.0 Nasal are not specifically only detecting the Omicron from the samples, but all SARS-CoV-2 regardless of the variants including the Omicron and its subvariants due to the modification on the binding ability of the antibody used in the RAT’s design. (**pages 4-5 lines: 81-92).**

However, as suggested by the reviewer, we added a discussion of the strengths of SARS-CoV-2 Rapid Antigen Test 2.0 and SARS-CoV-2 Rapid Antigen Test 2.0 Nasal in detecting Omicron compared to other RAT kits.

**Pages 18-19, Lines: 358-379**

5. Some studies claim currently available RAT kits are not affected by the emergence of the Omicron variant (PMID: 36519888, PMID: 36215709, PMID: 36798414, PMID: 35217106). Discussion can be provided to compare the claim of the current manuscript to gain a broader view of whether it is a real case that currently available RAT kits are not sensitive enough to the Omicron variant.

**Response:**

As suggested by the reviewer, we have added the discussion that comparing the currently available RATs that were reported are not affected by the emergence of the Omicron and the SARS-CoV-2 Rapid antigen test 2.0 and RAT 2.0 nasal.

**Pages: 18-19, Lines: 358-379.**

**“**The performance of RATs for the detection of SARS-CoV-2, particularly the Omicron and its subvariants is influenced by several factors, including the viral genetics and the design of the test devices. Hence, each RAT is affected differently by viral mutation due to the difference in the inherent design of each RAT. Apart from the studies that reported the decline of the RAT for Omicron detection, other studies claimed that some of the currently available RATs are not affected by the emergence of Omicron, particularly when being used to assess the samples with high viral load. The DIAGNOVIR SARS-CoV-2 ultra-rapid antigen test, for example, was reported to demonstrate high performance in detecting most SARS-CoV-2 variants including Wuhan, Alpha (B1.17), Beta (B.1.351), Delta (B.1.617.2), and Omicron (B.1.1529). A study on several currently available RAT kits demonstrated a similar level of sensitivity to Omicron and non-Omicron variants (On/Go, 76.4% and 71.0%; iHealth, 73.0%, and 71.0%; QuickVue, 84.3%, and 74.3%; BinaxNOW, 69.7%, and 71.0%; and InBios, 66.3% and 64.5%, respectively). However, most of these studies only evaluated the sensitivity and specificity for the detection of Omicron B.1.1529 but not for the detection of currently circulating subvariants. A study reported that most of the Omicron subvariants accumulated amino acid substitutions in the N protein. Given that the N protein is an important target protein for most of the currently available RATs, any mutation in this protein may affect the sensitivity of RATs. Hence, the SARS-CoV-2 Rapid Antigen Test 2.0 and SARS-CoV-2 Rapid Antigen Test 2.0 Nasal, which enable rapid and accurate detection of SARS-CoV-2, particularly the currently circulating Omicron subvariants such as BA.4 and BA.5, are considered promising to assist in the control of the person-to-person or closed-community transmission of these subvariants.”

6. WGS sequencing result was not available from the manuscript although authors declare Yes for all data are fully available without restriction.

**Response:**

As suggested by the reviewer, we have added the WGS sequencing result in the supplementary figure.

7. Line 48, HKUI can be converted to HKU-1.

**Response:**

We corrected it as recommended by the reviewer.

**Page 3, Line 47.**

8. Line 80-81, reference citation is required.

**Response:**

We have added the citation as suggested by the reviewer.

**Page 4, Lines 79-81.**

**Reviewer #2:**

This is a timely manuscript.

1. it is not audience friendly to place the figure legends under Materials and Methods.

**Response:**

We placed Figure 1 in the materials and methods to give a better picture of our study design. However, according to the journal requirement, during the submission, the figure should be submitted in a separate file, while the figure legends have to be placed close to the figure citation (page 6, line 105), that’s why in the current version (submitted version) of our manuscript, only figure legend appears under the Materials and Methods.

2. More details about the RT-PCR and WGS shall be provided, including commercial sources and Cat#.

**Response:**

We have added the catalog numbers for the RT-qPCR and the WGS system used in this study.

**Pages: 7-8, lines: 144-146, 159 and 168**
