# Supplementary material for: Efficacy of novel SARS-CoV-2 rapid antigen tests in the era of omicron outbreak
Source: PLoS One. 2023 Aug 10;18(8):e0289990. doi: 10.1371/journal.pone.0289990 (PMC10414561; doi:10.1371/journal.pone.0289990)
Supplement: Attachment — Submitted filename: Response to the reviewer.docx [file pone.0289990.s003.docx]

We would like to thank the reviewer and editor for their specific and helpful comments on the manuscript. We have carefully considered the comments and have revised the manuscript to address the reviewer and editor’s concerns. We have added a description of how the variant calling was used to identify the variants from the WGS data. Also, clarified and corrected the citation of Figure S1, so it won’t cause any misunderstanding. As per the journal requirements, we also double-checked the references list and confirmed that no cited papers have been retracted.

Detailed responses to the reviewer are reported as follows:

**Journal Requirements:**

Please review your reference list to ensure that it is complete and correct. If you have cited papers that have been retracted, please include the rationale for doing so in the manuscript text, or remove these references and replace them with relevant current references. Any changes to the reference list should be mentioned in the rebuttal letter that accompanies your revised manuscript. If you need to cite a retracted article, indicate the article’s retracted status in the References list and also include a citation and full reference for the retraction notice.

**Response:** We have checked all the references and confirmed that none of them have been retracted as mentioned, hence a kind reminder of which reference became a concern will greatly help us to revise it. Other than reference numbers: 25,26,27 that were added to answer the reviewer comments, no other changes were made to the reference list.

**Reviewer response:**

Reviewer #1:

Reply to response 1:
The hyperlinks list in references 24 and 25 might not be useful. Please revise the hyperlinks.

Response: As suggested by the reviewer, we have revised the hyperlinks for mentioned references.

**Reference 23 and 24.**


Reply to response 3:
The clarification is useful. Still, please provide a reference supporting the use of variant calling or describe how exactly it works to identify variants from WGS data.

Response:

The Omicron subvariants were determined through “variant calling” in the WGS analysis, which is the process by which the variants were identified according to the sequence data deposited in the database (GISAID). Briefly, the quality of the sequencing data in the format of Fastq was checked using the FastQC (v0.10.1) program, meanwhile, the Base Quality, Duplication level, and GC content were visually checked. Subsequently, the trimming procedure was employed to filter the reads to remove the low-quality sequences. The sickle (v.1.3.3) program was used for the entire filtering process. Further, the reads were aligned to the SARS-CoV-2 reference using BWA (B0.7.17) program, and the duplication reads generated through PCR were removed using GATK (v.4.0.2.1) program. Finally, the stand_call_conf - 30.0, stand_emit_conf -10.0, and dcov - 1,000 options were applied to find mutations within the sequence which already clear out from any duplication, and the variant analysis was performed using GATK (v.4.0.2.1). Further, the annotation of mutation from GATK (v.4.0.2.1) was finalized using the SnpEff (V.4.1) program and the SARS-CoV-2 strain of each sample was determined after comparing it with the sequences database in the global initiative for sharing all influenza data (GISAID, <https://gisaid.org/>) and with the mutation databases that were obtained from the lineage-specific mutation information database provided by the outbreak.info, CoVariants, and COVID CG.

**Pages: 8-9, Lines: 172-189**

Reply to response 6:

WGS data is provided. However, please provide more description for how Figure S1 could be co-referencing Table 4. Value for sample2 4, 21, 23, 28, and 46 look low. Are they representing negatively from the RAT tests? Possible to indicate Figure S1 for the RAT result?

Response:

Figure S1 represents the result of WGS hence do not directly represent the RAT test results. We have corrected the position where Figure S1 was being cited, so it won’t cause ambiguity or misunderstanding.

Additionally, among all samples assessed in this study, three were confirmed negative by RATs but positive by omicron PCR, one was confirmed negative by both RAT and Omicron PCR, and 52 samples were confirmed positive by both RAT and Omicron PCR. Therefore, for the WGS analysis, we assessed 55 samples, comprised of 52 samples positive by both RAT and Omicron PCR, and three samples confirmed positive by only Omicron PCR.

Among the samples 4,21,23,28, and 46 that have low values, only sample 4 was confirmed negative by RATs, hence the low values in WGS do not directly relate to the negativity of RAT. However, all these samples have low values which resulted in uncertainty of the lineage where the samples belong.

We also added some descriptions related to this issue in the WGS result section.

“We also compared the result from the RATs assay with that from WGS. In this study, we assessed 55 specimens for WGS, comprised of 52 confirmed positive by both RATs and Omicron PCR, and three confirmed positive by only Omicron PCR. One specimen was excluded from WGS analysis due to the negative result from both RATs and Omicron PCR tests.”

**Page 14, Lines: 265-269**

“The six specimens identified as “uncertain” showed a low value of the variables that were being assessed in the WGS analysis, hence making them difficult to be distinguished into specific lineages (S1 Fig). “

**Page 14, Lines: 277-279**
